# Uncarboxylated Osteocalcin Decreases SCD1 by Activating AMPK to Alleviate Hepatocyte Lipid Accumulation

**DOI:** 10.3390/molecules28073121

**Published:** 2023-03-31

**Authors:** Danqing Wang, Miao Zhang, Jiaojiao Xu, Jianhong Yang

**Affiliations:** Savaid Medical School, University of Chinese Academy of Sciences, Beijing 101400, China

**Keywords:** osteocalcin, lipid accumulation, SCD1, AMPK, signaling pathway

## Abstract

Uncarboxylated osteocalcin (GluOC), a small-molecule protein specifically synthesized and secreted by osteoblasts, is important in the regulation of energy metabolism. In our previous study, GluOC was shown to be effective in ameliorating dyslipidemia and hepatic steatosis in KKAy mice. However, the underlying mechanism of GluOC action on hepatocytes has not been well validated. In this study, oleic acid/palmitic acid (OA/PA)-induced HepG2 and NCTC 1469 cells were used as non-alcoholic fatty liver disease (NAFLD) cell models, and triacylglycerol (TG) levels were measured by oil red O staining, Nile Red staining, and ELISA. The fatty acid synthesis-related protein expression was detected by real-time quantitative polymerase chain reaction, Western blotting, and immunofluorescence. The results show that GluOC reduced triglyceride levels, and decreased the expression of sterol regulatory element-binding protein-1c (SREBP-1c) and stearyl-coenzyme A desaturase 1 (SCD1). si-SCD1 mimicked the lipid accumulation-reducing effect of GluOC, while overexpression of SCD1 attenuated the effect of GluOC. In addition, GluOC activated AMP-activated protein kinase (AMPK) phosphorylation to affect lipid metabolism in hepatocytes. Overall, the results of this study suggest that GluOC decreases SCD1 by activating AMPK to alleviate hepatocyte lipid accumulation, which provides a new target for improving NAFLD in further research.

## 1. Introduction

Non-alcoholic fatty liver disease (NAFLD) refers to a pathological disease of the liver caused by a combination of factors other than alcohol that damage the liver, and it is characterized by the accumulation and deposition of excessive fat in hepatocytes [1]. NAFLD is represented by a wide range of clinical case syndromes including steatosis and non-alcoholic steatohepatitis diseases and may further progress to cirrhosis [2], liver failure, and hepatocellular liver cancer, threatening human health [3]. NAFLD is the most common cause of liver disease worldwide [4], with multiple studies showing prevalence rates in the range of approximately 25% [5], and it is accompanied by increasing obesity and diabetes. In addition, NAFLD is also strongly associated with many diseases, including chronic liver disease, cardiovascular disease, thyroid disease, polycystic ovary syndrome, and colon cancer [6]. This disease has emerged as a significant public health concern that affects human health [7]. Therefore, in order to increase the effectiveness of illness control and the survival quality of patients, it is crucial to advance the study of this disease’s pathophysiology.

Osteocalcin (OC) is a non-collagenous bone matrix protein produced by osteoblasts [8], which can be γ-carboxylated at one or more of its glutamic acid residues [9]. Therefore, according to its degree of carboxylation, it can be divided into fully carboxylated osteocalcin and incompletely carboxylated osteocalcin, with incompletely carboxylated osteocalcin also including uncarboxylated osteocalcin (GluOC) [10]. OC is not only an indicator of bone health, but it also plays an important role in the regulation of glucose and lipid metabolism [11], male fertility [12], and cognitive performance of the brain [13] when it is present in the uncarboxylated form. It has been demonstrated that intraperitoneal administration of GluOC restores fatty liver degeneration in mice caused by a high-fat diet [14]. Our laboratory demonstrates that in KKAy mice, GluOC treatment enhances hepatic insulin signaling pathway, inhibits gluconeogenesis and promotes glycogen synthesis to improve hyperglycemia, and promotes fatty acid β-oxidation by inhibiting hepatic lipid ab initio synthesis to improve fatty liver and hypertriglyceridemia [15]. These studies suggest that GluOC has great potential for the treatment of the metabolic syndrome. However, the exact mechanism by which GluOC ameliorates NAFLD has not been fully elucidated.

Stearyl-coenzyme A desaturase 1 (SCD1) is a rate-limiting enzyme located on the endoplasmic reticulum for the synthesis of monounsaturated fatty acids [16], and can catalyze the introduction of the cis double bond in the Δ9 position of fatty acyl-CoA substrates to produce oleoyl-CoA (18:1) and palmitoyl-CoA (16:1) [17], the main components of synthetic triglyceride (TG) [18]. It has been shown that SCD1 knockout or liver-specific SCD1 knockout mice present increased expression of fatty acid oxidation-related genes and decreased expression of key adipogenic genes, resulting in decreased triglyceride synthesis and secretion [19]. This suggests that SCD1 is involved in the pathophysiology of nonalcoholic fatty liver. Our laboratory demonstrated that GluOC treatment ameliorates hepatic steatosis by inhibiting de novo lipogenesis and enhancing fatty-acid β-oxidation in KKAy mice. However, whether the beneficial effects of GluOC are related to SCD1 and the related signaling pathways needs to be further investigated.

AMPK signaling is critical in regulating lipid metabolism in hepatocytes [20]. Several studies have shown that enhanced AMPK activity is thought to alleviate NAFLD [21]. AMPK can regulate sterol regulatory element binding protein-1c (SREBP-1c), a crucial transcription factor related to lipid metabolism [22]. SREBP-1c has also been shown to have regulatory effects on genes, including fatty acid synthases, such as acetyl-CoA carboxylase 1 (ACC1), fatty acid synthetase (FAS), and SCD1.

G-protein-coupled receptor, class C, group 6, subtype A (GPRC6A), a relatively recently discovered G-protein-coupled receptor, is activated by multiple ligands, including osteocalcin, testosterone, basic amino acids, and various cations [23]. GPRC6A has been identified in adipocytes, testicular mesenchymal cells, and pancreatic islet cells in response to GluOC and increased the production and release of testosterone, lipocalin, and insulin [24]. However, whether GPRC6A acts as a GluOC receptor in HepG2 has not yet been confirmed, at least to the best of our knowledge.

In this study, on the basis of previous results, we show that GluOC decreases SCD1 by activating AMPK to alleviate hepatocyte lipid accumulation, and we also demonstrate that GPRC6A is the receptor for GluOC in HepG2 cells

## 2. Results

### 2.1. GluOC Inhibits Lipid Accumulation in Oleic Acid (OA)- and Palmitic Acid (PA)-Induced HepG2 and NCTC 1469 Cells

**Figure 1:**
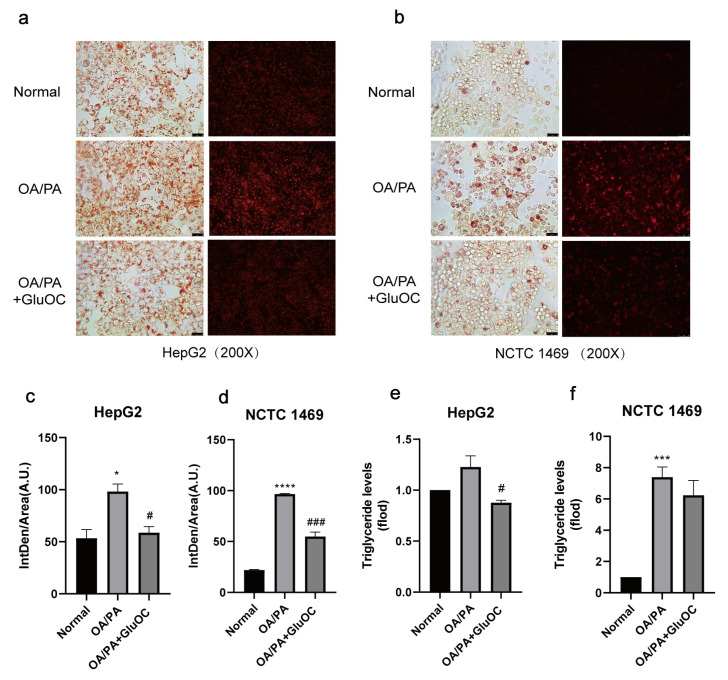
GluOC improved OA/PA-induced lipid accumulation and triglyceride synthesis in hepatocytes. (**a**,**b**) Representative photomicrographs for oil red O staining and Nile Red straining. For OA/PA steatosis cell model, the HepG2 and NCTC 1469 cells were treated with or without 18 ng/mL GluOC for 24 h in serum-free media. (**c**,**d**) Mean fluorescence intensity of Figure 1a,b. (**e**,**f**) OA/PA increased the TG accumulation in HepG2 cells (**e**) and NCTC 1469 cells (**f**), and treatment with GluOC decreased the TG accumulation in these cells. The data are presented as the mean ± SE of three independent experiments. * *p* < 0.05, *** *p* < 0.001, and **** *p* < 0.0001 versus Normal group; # *p* < 0.05 and ### *p* < 0.001 versus OA/PA group. OA/PA, oleic acid, and palmitic acid; GluOC, OA/PA + uncarboxylated osteocalcin.

In order to investigate the effect of GluOC on lipid accumulation in OA/PA-induced HepG2 and NCTC 1469 cells, we monitored the intracellular lipid droplets after GluOC treatment by staining them with oil red O, and Nile Red. As shown in Figure 1a–d, compared with the normal group, lipid accumulation was significantly increased in the OA/PA-induced group, proving the successful construction of our lipid accumulation model. In addition, the lipid accumulation was significantly reduced after adding GluOC. Furthermore, the triglyceride assays showed that TG content increased in the model group compared to the control group, while intracellular TG content decreased after GluOC treatment (Figure 1e,f). This indicates that GluOC inhibits OA/PA-induced TG accumulation in HepG2 and NCTC 1469 cells.

To simplify the subsequent experiments, we denoted the OA/PA-induced group as the control group and the GluOC-treated group as the experimental group.

### 2.2. GluOC Decreases the Expression of Genes Related to the De Novo Lipogenesis

**Figure 2:**
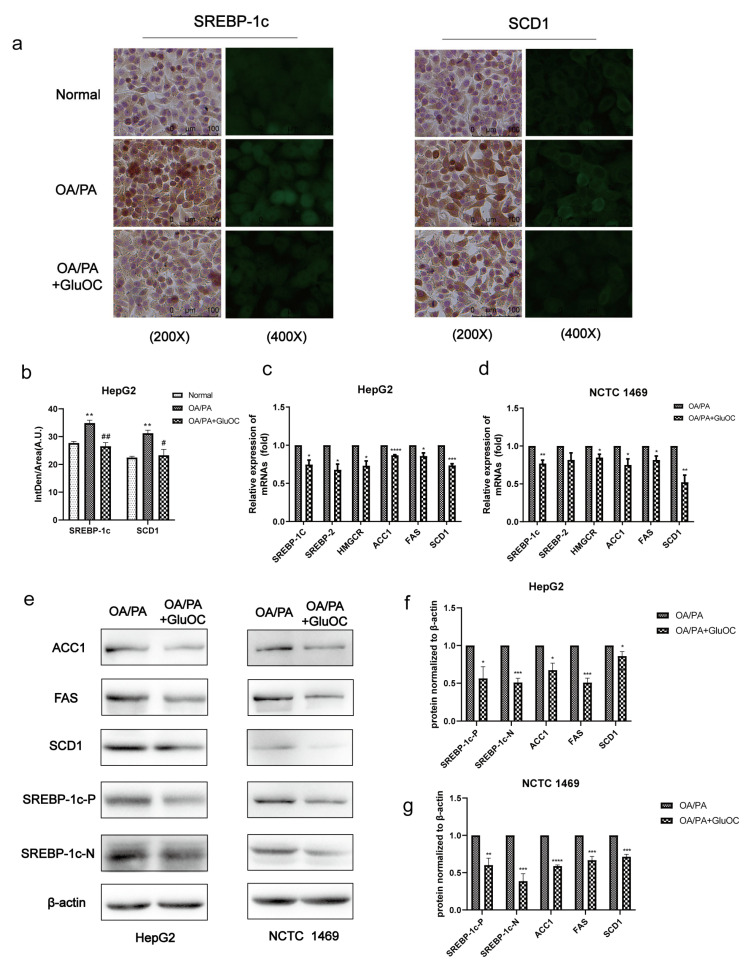
GluOC reduced OA/PA-induced high expression of genes associated with TG de novo synthesis in hepatocytes. (**a**) Immunohistochemistry (IHC) and immunofluorescence (IF) experiments demonstrated that GluOC significantly decreased the expression of SREBP-1c and SCD1. (**b**) Mean fluorescence intensity of Figure 2a. The data are presented as the mean ± SD of three independent experiments. ** *p* < 0.01 versus Normal group; # *p* < 0.05, ## *p* < 0.01 versus OA/PA group. (**c**,**d**) GluOC decreased OA/PA-induced mRNA expression of SREBP-1c, SREBP-2, HMGCR, SCD1, FAS, and ACC1 in HepG2 cells and NCTC 1469 cells. (**e**–**g**) Compared with the OA/PA group, GluOC decreased the protein expression of SREBP-1c-N, SREBP-1c-P, SCD1, FAS, and ACC1 in HepG2 cells and NCTC 1469 cells. Their densitometric quantification was normalized to β-actin. The data are presented as the mean ± SE of three independent experiments. * *p* < 0.05, ** *p* < 0.01, *** *p* < 0.001, and **** *p* < 0.0001 versus OA/PA group. SREBP-1c-P, SREBP-1c precursor; SREBP-1c-N, SREBP-1c nuclear active forms; OA/PA, oleic acid and palmitic acid; GluOC, OA/PA + uncarboxylated osteocalcin.

SREBP-1c and SCD1 play critical roles in fatty acid synthesis. We found that the expression of SREBP-1c and SCD1 was significantly increased in the OA/PA group compared with the normal group, and the expression of both was inhibited again after GluOC treatment (Figure 2a,b). Subsequently, we characterized the expression of genes associated with de novo lipogenesis in HepG2 and NCTC 1469 cells (Figure 2c,d). Sterol regulatory element-binding protein 2 (SREBP2) and anti-3-hydroxy-3-methylglutaryl-coenzyme A reductase (HMGCR) are both cholesterol synthesis-related genes, and SREBP-1c, ACC1, FAS, and SCD1 are triglyceride synthesis-related genes, and their mRNA levels were all reduced after GluOC treatment. We also detected their protein levels, which matched the changes in mRNA. Compared with the OA/PA group, GluOC inhibited the expression of the precursor of SREBP-1c (SREBP-1c-P) and its mature forms (SREBP-1c-N) in the nucleus. SREBP-1c is thought to be an acritical transcription factor in regulating the de novo synthesis of lipids in the liver [25]. Generally, such research results suggest that GluOC inhibits the OA/PA-induced expression of SCD1 and other genes associated with the de novo lipogenesis in vitro (Figure 2e–g). It also shows that GluOC can effectively alleviate the lipid accumulation in hepatocytes induced by OA/PA.

### 2.3. SCD1 Mediates the Alleviation of OA/PA-Induced Lipid Accumulation by GluOC

We further investigated whether SCD1 mediates the effect of GluOC on OP/PA-induced lipid accumulation, mainly by Nile Red staining and triglyceride assays.

**Figure 3:**
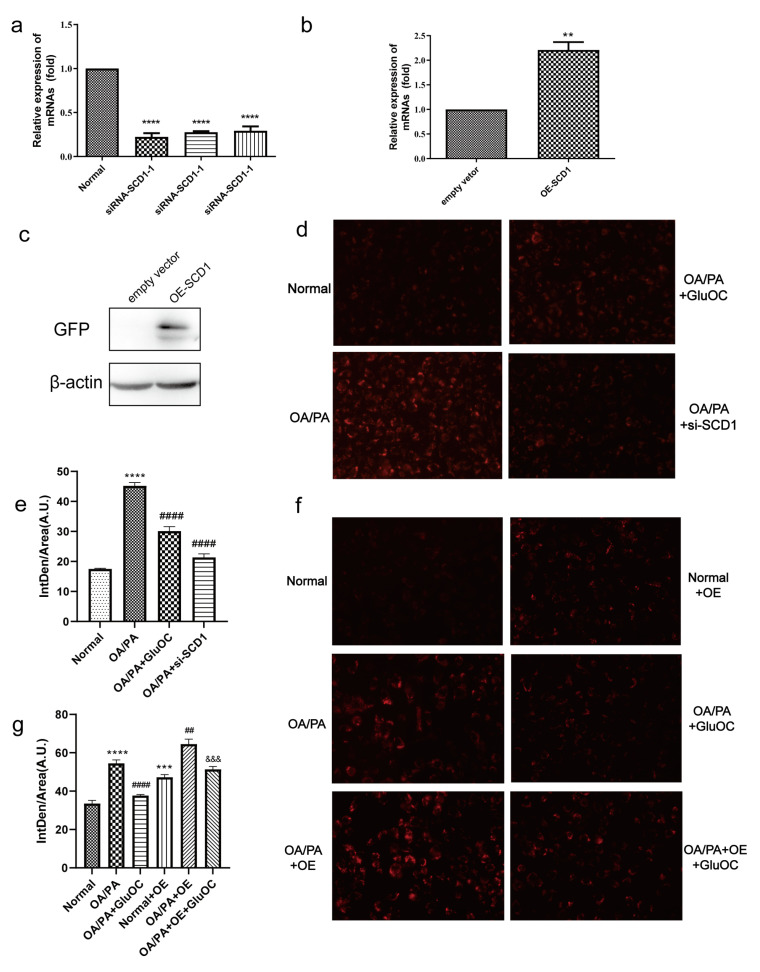
SCD1 mediated the effect of GluOC in attenuating the OA/PA-induced TG accumulation in HepG2 cells. (**a**) siRNA-SCD1-1, siRNA-SCD1-2 and siRNA-SCD1-3, all significantly suppressed the expression of SCD1. (**b**,**c**) Verification of the overexpression of SCD1. (**d**) The knockdown of SCD1 mimicked the effect of GluOC on decreasing TG levels in HepG2 cells exposed to OA/PA. (**e**) Mean fluorescence intensity of Figure 3d. (**f**) The overexpression of SCD1 attenuated the effect of GluOC on decreasing TG accumulation in HepG2 cells exposed to OA/PA. (**g**) Mean fluorescence intensity of Figure 3f. The data are presented as the mean ± SE of three independent experiments. ** *p* < 0.01, *** *p* < 0.001, and **** *p* < 0.0001 versus Normal Group; ## *p* < 0.01, and #### *p* < 0.0001 versus OA/PA group; &&& *p* < 0.001 versus OA/PA + OE group. OA/PA + si-SCD1, oleic acid and palmitic acid + siRNA-SCD1-2; OE-SCD1, overexpression of SCD1; Normal + OE, Normal + overexpression of SCD1; OA/PA + OE, oleic acid and palmitic acid + overexpression of SCD1; OA/PA + OE + GluOC, oleic acid and palmitic acid + overexpression of SCD1 + uncarboxylated osteocalcin.

We first inhibited SCD1 expression in HepG2 cells. As shown in Figure 3a, in HepG2 cells transfected with siRNA-SCD1-1, siRNA-SCD1-2, and siRNA-SCD1-3, they all significantly suppressed the expression of SCD1, and siRNA-SCD1-2 was selected for use in the following experiments. Moreover, to further verify the relationship between GluOC and SCD1, we overexpressed SCD1 (Figure 3b,c). We found that siRNA-SCD1 reduced the increase in intracellular accumulation of lipid droplets induced by OA/PA. This suggests that inhibition of SCD1 expression reduces OA/PA-induced hepatocyte steatosis (Figure 3d,e). The knockdown of SCD1 mimicked the effect of GluOC on decreasing TG levels in OA/PA-induced HepG2 cells. The Nile Red staining revealed that overexpression of SCD1 (OE-SCD1) increased lipid accumulation, and attenuated the effect of GluOC decreasing lipid accumulation. Meanwhile, GluOC also decreased the TG accumulation in HepG2 cells overexpressing SCD1 and exposed to OA/PA (Figure 3f,g). These results indicate that SCD1 plays a critical role in the pathophysiology of lipid accumulation in HepG2 cells.

### 2.4. GluOC Activates the Phosphorylation of AMPK (Thr172) in OA/PA-Induced HepG2 Cells

**Figure 4:**
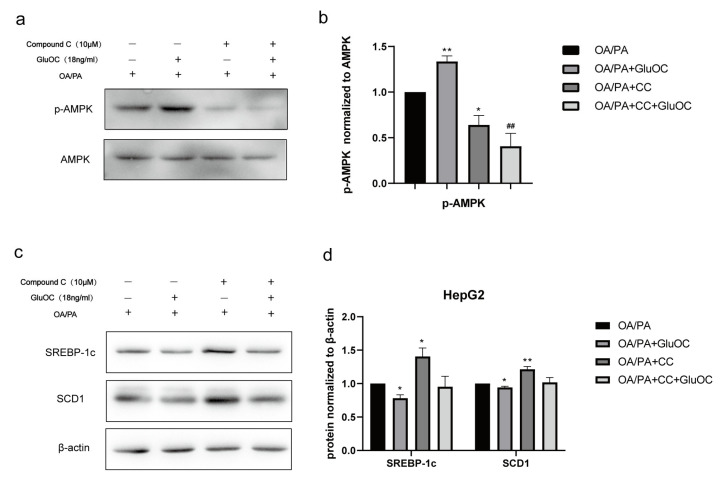
The AMPK pathway mediated the effect of GluOC in reducing SREBP-1c and SCD1 expression. (**a**,**b**) GluOC promotes the phosphorylation of AMPK (Thr172) in vitro. The densitometric quantification of p-AMPK is normalized to AMPK. (**c**,**d**) The inhibition of AMPK phosphorylation attenuated the effect of GluOC in decreasing SREBP-1c and SCD1 expression in HepG2 cells. The densitometric quantification of SREBP-1c and SCD1 is normalized to β-actin. The data are presented as the mean ± SE of three independent experiments. * *p* < 0.05, and ** *p* < 0.01 versus OA/PA group. ## *p* < 0.01 versus OA/PA + GluOC group. OA/PA + CC, oleic acid, and palmitic acid + Compound C; OA/PA + CC + GluOC, oleic acid, and palmitic acid + Compound C + uncarboxylated osteocalcin.

In order to reveal the mechanism of GluOC in inhibiting lipid accumulation in OA/PA-induced HepG2 cells, we measured the expression of AMPK and p-AMPK by Western blotting after treatment with or without GluOC. Figure 4a,b indicated that phosphorylation of AMPK (Thr 172) increased in HepG2 cells after the treatment with GluOC.

### 2.5. GluOC Decreases SREBP-1c and SCD1 Expression by Activating AMPK

Furthermore, we performed another experiment using the AMPK inhibitor Compound C (CC). CC was preincubated with HepG2 cells for 1 h to inhibit the activation of AMPK in advance, and then, HepG2 cells were treated with OA/PA and GluOC for another 24 h. The protein expression levels of SREBP-1c and SCD1 were detected by Western blotting. The results revealed that inhibiting the phosphorylation of AMPK (Thr172) could improve the expression of SREBP-1c and SCD1 in HepG2 cells. Furthermore, GluOC’s ability to inhibit the expression of SREBP-1c and SCD1 became weak along this process (Figure 4c,d).In conclusion, the experiments reveal that GluOC decreases SREBP-1c and SCD1 expression by activating AMPK.

### 2.6. GPRC6A Is a Potential Receptor for GluOC

**Figure 5:**
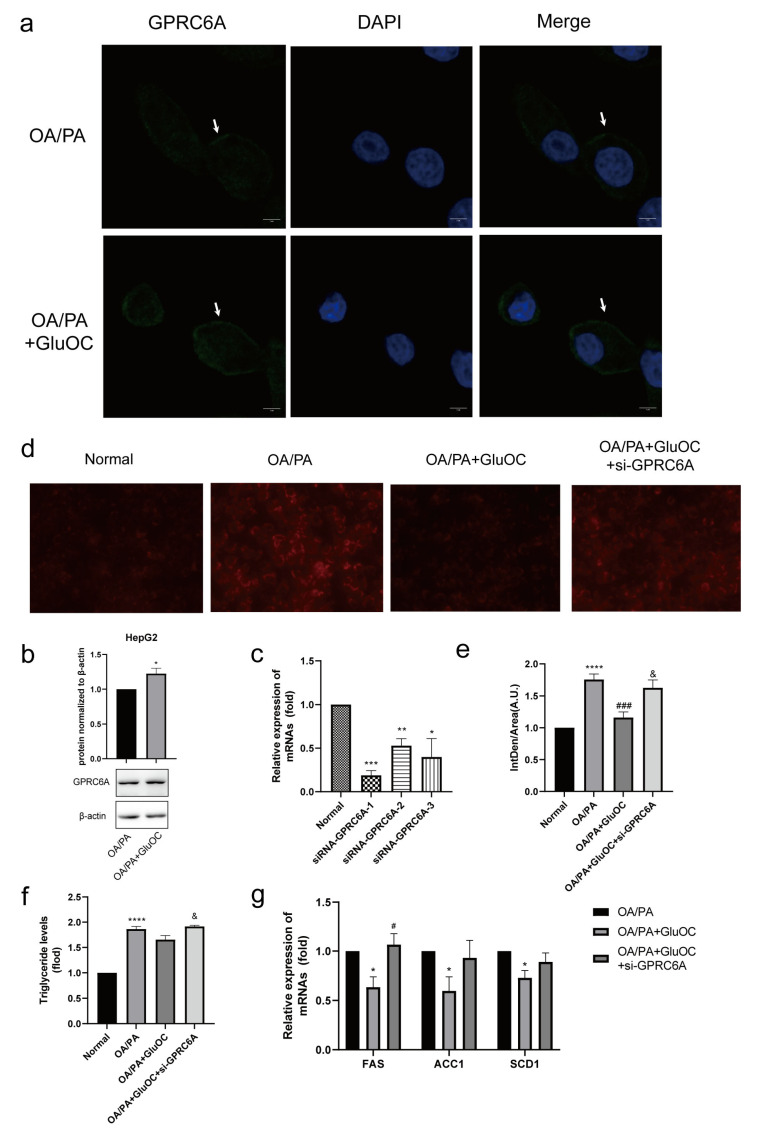
GPRC6A is a potential receptor for GluOC. (**a**) The expression of GPRC6A was detected by immunofluorescence, and its fluorescence intensity increased after the addition of GluOC. (**b**) It showed that the expression of GPRC6A increased after the addition of GluOC by Western blotting. GPCR6A is normalized to β-actin. The data are presented as the mean ± SE of three independent experiments. * *p* < 0.05 versus OA/PA group. (**c**) siRNA-GPRC6A-1, siRNA-GPRC6A-2 and siRNA-GPRC6A-3, all significantly suppressed the expression of GPRC6A. (**d**) The effects of GluOC alleviated hepatocyte lipid accumulation were reversed by the knockdown of GPRC6A. (**e**) Mean fluorescence intensity of Figure 5d. (**f**) The effects of GluOC alleviated TG levels were reversed by the knockdown of GPRC6A. (**g**) mRNA expression of the triglyceride synthesis-related genes (FAS, ACC1, and SCD1). The data are presented as the mean ± SD of three independent experiments. * *p* < 0.05, ** *p* < 0.01, *** *p* < 0.001, and **** *p* < 0.0001 versus Normal group; # *p* < 0.05, ### *p* < 0.001 versus OA/PA group; & *p* < 0.05 versus OA/PA + GluOC group. OA/PA + GluOC + si-GPRC6A, oleic acid and palmitic acid + uncarboxylated osteocalcin+ siRNA-GPRC6A-1.

In contrast to the control group, we discovered that the expression of GPRC6A increased after adding GluOC (Figure 5a,b). To further verify the function of GPRC6A in cells, siRNAs were used to knockdown GPRC6A expression. RT-qPCR analysis indicated that siRNA-GPR6CA-1 significantly reduced the expression of GPRC6A (Figure 5c). Based on these results, siRNA-GPR6CA-1 was selected to knockdown GPRC6A expression in all subsequent experiments. In order to reveal the effects of GPRC6A in HepG2 cells, the study examined the lipid accumulation following GPRC6A knockdown (Figure 5d,e). The effects of GluOC alleviating hepatocyte lipid accumulation were reversed by the knockdown of GPRC6A (Figure 5f). Similarly, the down-regulation of lipid synthesis-related genes (FAS, ACC1, and SCD1) by GluOC were reversed after the knockdown of GPRC6A, the results were similar to those in the OA/PA group (Figure 5g). In summary, these results suggest that GPRC6A functions as a potential receptor of GluOC in HepG2 cells. 

### 2.7. Molecular Docking Was Used to Simulate the Interaction Type of GluOC and GPRC6A

**Figure 6:**
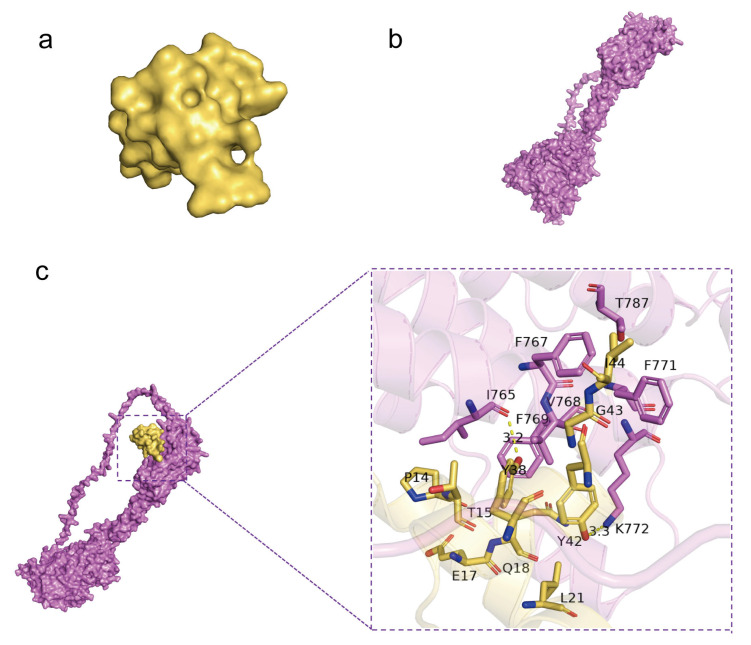
Molecular docking by means of computer simulation proved that GluOC acted on the 7tm_GPCRs superfamily of GPRC6A. (**a**) Protein structure of GluOC. (**b**) Protein structure of GPRC6A. (**c**) Computational prediction of the docking site of GluOC and GPRC6A. I 765 and K 772 of GPRC6A form hydrogen bonds with Y38 and Y42 of GluOC, and the lengths of the hydrogen bonds are 3.2 Å and 3.3 Å.

Molecular docking by means of computer simulation proves that GluOC acts on the 7tm_GPCRs superfamily of GPRC6A. We chose the best-scoring pose for the picture (Figure 6a,b). Ile 765 and Lys 772 of GPRC6A form hydrogen bonds with Tyr 38 and Tyr 42 of GluOC, and the lengths of hydrogen bonds are 3.2 Å and 3.3 Å (Table 1). F769, V768, F767, T787, and F771 of GPRC6A have hydrophobic interactions with I44, G43, T15, P14, E17, Q18, and L21 of GluOC, and these residues together make up both proteins’ hydrophobic surfaces (Figure 6c).

## 3. Discussion

**Figure 7:**
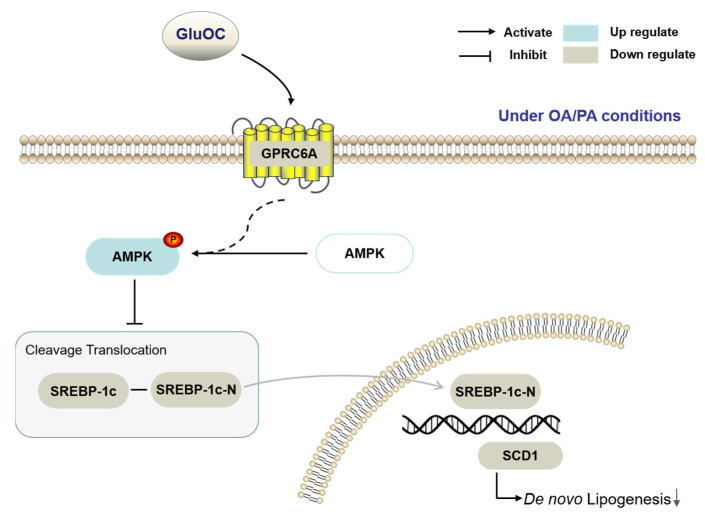
The working hypothesis of the effects of GluOC on the HepG2 cells under OA/PA conditions.

The present experimental study demonstrates that GluOC decreases SCD1 by activating AMPK to alleviate hepatocyte lipid accumulation (Figure 7). GluOC promotes phosphorylation of AMPK (Thr172) and inhibits SREBP-1c moving into the nucleus of HepG2 cells, resulting in reduced SCD1 expression and decreased TG accumulation in hepatocytes.

NAFLD encompasses a continuum of diseases from steatosis with or without mild inflammation to non-alcoholic steatohepatitis (NASH). Hepatocytes have a significant buildup of lipids, which leads to NAFLD [26]. Naturally, symptoms of NAFLD could be alleviated by reducing lipid accumulation and inflammation. GluOC is a small-molecule peptide that exists in the body as a hormone with many pharmacological effects, including lowering TG and cholesterol levels in animal liver and serum [27]. Therefore, comprehending the mechanism of GluOC in hepatocytes will assist in the development of GluOC as a therapeutic agent for NAFLD.

There is a dynamic equilibrium between lipid acquisition (free fatty acid uptake or de novo lipogenesis) and lipid clearance (oxidation or assembly and secretion of very low-density lipoprotein). When this balance is disturbed, it leads to lipid accumulation. In NAFLD patients, de novo lipogenesis contributes 26.1 ± 6.7% to the lipid storage resources in the liver [28]. Oleic and palmitic acids make up the majority of the free fatty acids in the livers of NAFLD patients, so an overabundance of lipids causes steatosis [29]. As a result, in a model of NAFLD, oleic and palmitic acids are frequently used as stimulating agents to activate hepatocytes [30]. In the present study, we used 250 µM oleic acid and 125 µM palmitic acid to co-stimulate lipid accumulation in human HepG2 cells and mouse primary hepatocytes NCTC 1469, as shown in Figure 1, and lipid accumulation in hepatocytes is significantly increased by the stimulation. This indicates that the lipid accumulation model had been successfully established.

Osteocalcin, a non-collagenous bone matrix protein produced by osteoblasts, has been reported to modulate glucose and lipid metabolism when administered in a non-carboxylated form [31]. We find that GluOC improves OA/PA-induced lipid metabolism in hepatocytes and reduces the accumulation of TG (Figure 1). Additionally, GluOC decreases the expression of SCD1 in model cells. In contrast, it has been shown that SCD1 catalyzes the synthesis of monounsaturated fatty acids, which are the main substrate for TG [17]. A defective SCD1 gene leads to resistance to diet-induced obesity [32], increased insulin sensitivity, and increased metabolic rate [33]. Thus, SCD1 plays a key role in the pathophysiology of TG accumulation in the liver [34]. The present study shows that the knockdown of SCD1 in OA/PA-induced hepatocytes reduces lipid accumulation, which is consistent with the effect of adding GluOC. In contrast, overexpression of SCD1 results in increased lipid accumulation and attenuates the effect of GluOC in reducing lipid accumulation, suggesting that SCD1 mediates the effect of GluOC in reducing lipid accumulation in hepatocytes (Figure 3). 

SREBP-1c, an upstream of SCD1 [35], is a critical nuclear transcription factor that regulates the expression of genes related to fatty acid synthesis in vivo [36]. The results show, the mRNA and protein expression levels of SREBP-1c could be greatly reduced with GluOC treatment, so it can be concluded that GluOC may improve lipid accumulation by affecting the SREBP-1c/SCD1 signaling pathway. The SREBP-1c in Figure 2 contains both precursor and mature forms. SREBP-1c is synthesized as an inactive precursor, undergoes proteolytic cleavage and maturation in the Golgi apparatus, and then mature SREBP-1c migrates to the nucleus to activate the transcription of target genes. Our study shows that protein expression of both precursor and mature forms of SREBP-1c is reduced after GluOC treatment [37]. The changes in both forms are in parallel [38]. Thus, the reduced expression of SREBP-1c can be considered as down-regulating the expression of its downstream gene SCD1, thus reducing lipid biosynthesis and alleviating NAFLD. In addition to SREBP-1c and SCD1, GluOC also regulates the expression of other genes related to TG anabolism. ACC1 promotes adenosine triphosphatase-dependent carboxylation of acetyl coenzyme A to malonyl coenzyme A [39]. This is the first rate-limiting step in de novo lipogenesis. FAS sequentially uses malonyl coenzyme a to extend the growing fatty acyl chain by two carbons to form saturated palmitate, the major product of fatty acid synthesis [40]. They are both regulated by SREBP-1c. These genes are important in the fatty acid synthesis pathway and are closely associated with the development of NAFLD [41]. Therefore, GluOC might improve NAFLD by targeting multiple genes associated with lipid metabolism.

AMPK is a kinase that improves energy metabolism by regulating lipid catabolism and anabolism, it is a potential therapeutic target for metabolic diseases because it regulates the expression of genes related to energy metabolism and therefore plays an important role in energy metabolism [42,43]. Its phosphorylated form is involved in the regulation of lipid metabolism [44]. It has been demonstrated that MTL (the active segment of OC) stimulates AMPK activation in hepatocytes and subsequently inhibits downstream ACC1 activation [45]. In line with these data, our study shows that in HepG2 cells, phosphorylated AMPK increases after GluOC treatment. Furthermore, we find that inhibition of AMPK activation increases SREBP-1c and SCD1 expression in HepG2 cells, suggesting that AMPK mediates the effect of GluOC in reducing SCD1 expression. GluOC inhibits OA/PA-induced ab initio adipogenesis in hepatocytes by activating the AMPK–SREBP-1c–SCD1 signaling pathway (Figure 4). Therefore, treatment with GluOC may prevent and improve NAFLD, but further studies are needed to verify its efficacy and safety in vivo. 

GPRC6A, a widely expressed G-protein coupled receptor, is proposed to be a master regulator of complex endocrine networks and metabolic processes [46]. Our previous work has demonstrated that osteocalcin can activate AMPK through the putative receptor GPRC6A, thereby promoting the differentiation of pro-osteoblasts [47]. In the present experiments, we confirm that GPRC6A expression increases after the addition of GluOC in OA/PA-induced HepG2 cells. The effects of GluOC alleviated hepatocyte lipid accumulation are reversed by the knockdown of GPRC6A (Figure 5). The mRNA expression of FAS, ACC1, and SCD1 is also reversed, the results are similar to those in the OA/PA group These results suggest that GPRC6A functions as a potential receptor of GluOC in HepG2 cells. We also identify the pose with the greatest score through computer molecular docking simulation (Figure 6). These results showed that GPRC6A is a potential receptor of GluOC. However, more research is still required to understand how GluOC and GPRC6A work thoroughly.

## 4. Materials and Methods

### 4.1. Reagents and Antibodies

Oil Red O, oleic acid, and palmitic acid were purchased from Sigma-Aldrich (St. Louis, MO, USA). Nile Red was purchased from MedChemExpress (Monmouth Junction, NJ, USA). Compound C (AMPK inhibitor) was purchased from Selleck (Houston, TX, USA). The BCA protein concentration assay kit was purchased from Lablead Biotech (Beijing, China). Antibodies against FAS (3180s), AMPKα (5831), and β-actin (4970) were purchased from Cell Signaling Technology (Danvers, MA, USA). Antibodies against ACC1 (67373-1-Ig), SREBP1 (14088-1-AP), and SCD1 (23393-1-AP) were purchased from Proteintech Group (Chicago, IL, USA). Antibodies against SCD1 (ab236868) and phospho-AMPKα (ab133448) were purchased from Abcam (Cambridge, MA, USA). Antibody against GPRC6A was purchased from ABclonal. Antibodies against goat anti-rabbit IgG (H+L)-HRP and goat anti-mouse IgG (H+L)-HRP were purchased from Lablead Biotech (Beijing, China). GluOC was obtained from prokaryotic expression and purification in our laboratory.

### 4.2. Cells and Cell Culture

HepG2 cells were obtained from The Cell Bank of Type Culture Collection of The Chinese Academy of Sciences. Initially, the cells were grown in Dulbecco’s modified Eagle’s medium (DMEM, Gibco, Carlsbad, CA, USA), containing 10% fetal bovine serum (FBS, Gibco, Carlsbad, CA, USA). NCTC 1469 cells were obtained from Procell Life Science&Technology. Firstly, the cells were grown in Dulbecco’s modified Eagle’s medium (DMEM, Procell, Wuhan, China), containing 10% Donor Equine serum (HS, Procell). After that, the cells were cultured in a moist 37 °C incubator, with 5% CO_2_. For the in vitro experiments, cells were treated with or without GluOC in serum-free media containing 250 µM oleic acid (OA) and 125 µM palmitic acid (PA) for 24 h. We chose 18 ng/mL GluOC in cell experiments based on our previous study. In the inhibitor assay, when cells were confluent to 80%, HepG2 cells were first treated with CC for 1 h and then treated with or without GluOC in serum-free media containing 250 µM oleic acid (OA) and 125 µM palmitic acid (PA) for 24 h.

### 4.3. Cell Staining

The samples were washed three times with PBS and then fixed with 4% polyformaldehyde (Leagene, Beijing, China) for 15 min. Then, the cells were treated with 0.5% Triton X-100 for 15 min and were finally stained with oil red O work solution (oil red O stock solution: distilled water = 3:2) and Nile Red work solution at room temperature for 60 min. Following staining, cells were washed three times with PBS after the removal of excess dye and then pictured using an optical microscope at 200× magnification (Leica Microsystems, Wetzlar, Germany).

### 4.4. Measurement of the Triacylglycerol (TG) Level

After the treatment of HepG2 cells, cell samples were resuspended in PBS and measured by using an ELISA kit (AB-J0604A; Abmart Shanghai Co., Ltd., Shanghai, China) according to the manufacturer’s instructions. In addition, for NCTC 1469 cells, the TG concentration was measured using the TG reagent kit (E1013; Applygen Technologics Inc., Beijing, China) according to the manufacturer’s protocol.

### 4.5. Transfection Assay

Interfering fragments (si-SCD1 and si-GPRC6A) were designed and produced by Sangon Biotech (Shanghai) Co., Ltd. (Shanghai, China). The si-SCD1 sequences were as follows: siRNA-SCD1-1 sense: 5′-CGUCCUUAUGACAAGAACAUUTT-3′; siRNA-SCD1-2 sense: 5′-CUACGGCUCUUUCUGAUCAUUTT-3′; siRNA-SCD1-3 sense: 5′-CCCACCUACAAGGAUAAGGAATT-3′; The si-GPRC6A sequences were as follows: siRNA-GPRC6A-1 sense: 5′-CCACAAAUCCAGGAGUGUGUUTT-3′; siRNA-GPRC6A-2 sense: 5′-GAAGCAAAUAACGUGUGCAUATT-3′; siRNA-GPRC6A-3 sense: 5′-GCUGUGGAGAUUAUUGUCAUATT-3′; negative control siRNA sense: 5′-UUCUCCGAACGUUGCACGUTT-3′. SCD1. Overexpression (SCD1-OE) was generated by the Plvx-CMV-SCD1-EGFP vector. The CDS sequence (NM_005063.5) of the SCD1 gene was obtained from NCBI, and the SCD1 fragment was amplified from the cDNA of HepG2 cells using Phanta Max Super-Fidelity DNA Polymerase (P505). ClonExpress Ultra One Step Cloning Kit (C115) was used to clone the SCD1 fragment and the EGFP fragment into the plvx vector (xho I, xba I double enzyme cleavage). The fragments were mixed with Lipofectamine 3000 (Thermo Fisher Scientific, Waltham, MA, USA) in a solution of opti-MEM (Gibco, Carlsbad, CA, USA). After incubation for 20 min, the solution was added to the cell medium. After 24 h, we changed the medium to basal medium or other different treatments. After 48 h, the cells were collected for testing.

### 4.6. Reverse Transcription–Quantitative PCR (RT–qPCR)

Total RNA extraction from the HepG2 and NCTC 1469 cells was performed using a Total RNA kit (DP419; Tiangen Biotech, Beijing, China). A total of 2 µg RNA was reverse transcribed into cDNA using the TransScript One-Step gDNA Removal and cDNA Synthesis SuperMix (AT311-03; TransGen Biotech, Beijing, China) and quantitative PCR (qPCR) was performed in a 20 µL reaction volume with the TransStart Top Green qPCR SuperMix (+Dye II) (AQ132-24; TransGen Biotech, Beijing, China). The following thermocycling conditions were used: 40 cycles at 94 °C for 5 s, 60 °C for 15 s, and 72 °C for 10 s. The sequences of the primers used are listed in Table 2. Expression data were normalized to GAPDH and β-actin, which was used as the internal standard.

### 4.7. Western Blotting Assay

Total protein was extracted with a RIPA lysis buffer system (R0010, Solarbio Science&Technology, Beijing, China) according to the manufacturer’s protocol. The protein concentrations of the extracted proteins were measured using a BCA Detection kit (B5000; Lablead Biotech, Beijing, China). A total of 30 µg of proteins (for each lane) in cell lysates were loaded and separated by 10% SDS-PAGE and then transferred to PVDF membranes. The membranes were blocked in TBS-5% Tween-20 (TBST) containing 5% non-fat milk at room temperature for 2 h and incubated overnight at 4 °C with antibodies. The membranes were then washed three times with TBST (10 min each), followed by incubation with horseradish-peroxidase-conjugated secondary antibody (1:5000; Y1055; Beijing Lablead Biotech) at 25 °C for 1 h. The protein bands were visualized using an ECL kit (170-5060; BioRad, Hercules, CA, USA). To quantify the expression of proteins, the intensity of bands was normalized to the respective β-actin control, and the bands of the phospho-proteins were normalized to the total levels of that specific protein. ImageJ version 6 (National Institutes of Health, Bethesda, MD, USA) was used for densitometric analysis.

### 4.8. Immunohistochemistry (IHC) and Immunofluorescence (IF)

Cells were inoculated in advance on the circular microscope cover glasses, fixed in 4% paraformaldehyde for 15 min, washed three times with PBS, then incubated with 0.5% Triton-X-100 for 15 min, washed three times with PBS, followed by antigen repair with 20 ug/mL proteinase K for 30 min, then incubated with 3% hydrogen peroxide-methanol mixture for 5 min at room temperature, and washed three times with PBS. Next, the antigen was closed with 10% BSA for 30 min and washed three times with PBS. The samples were added and incubated overnight for 4 °C with antibodies and washed three times with PBS the next day. The secondary antibody (33116ES60, Yeasen, Shanghai, China) was incubated for 1 h at 37 °C and washed three times with PBS. For IF, it was treated with an antifade mounting medium containing DAPI (ZLI-9557, ZSGB-BIO, Beijing, China) before observation. In addition, it was then pictured using an optical microscope at 400× magnification (Leica Microsystems, Wetzlar, Germany; and Carl Zeiss LSM880, Jena, Germany).

### 4.9. Molecular Docking Simulation of GluOC with GPRC6A

Docking is particularly useful in studying drug target discovery. In this project, Z-DOCK was used to explore the binding affinity between GluOC and GPRC6A, and the GPRC6A structure was downloaded from the PDB database.

### 4.10. Statistical Analysis

Data are presented as the means ± standard error of at least three independent repetitions. Statistical analysis was performed using GraphPad Prism version 9.0 (GraphPad Software, Inc., La Jolla, CA, USA). Statistical differences between the two groups were determined using a two-tailed Student’s t-test, while statistical differences among multiple groups were evaluated using a one-way ANOVA followed by Tukey’s post hoc test. A *p* < 0.05 was considered to indicate a statistically significant difference.

## 5. Conclusions

In this study, OA/PA-induced HepG2 and NCTC 1469 cells were used as NAFLD cell models. The addition of GluOC significantly inhibited hepatocyte lipid accumulation and activated the phosphorylation of the signaling pathway protein AMPK. The down-regulation effect of GluOC on SREBP-1c and SCD1 expression was reversed by the addition of an AMPK inhibitor. Meanwhile, the knockdown of SCD1 expression mimicked the lipid accumulation-reducing effect of GluOC, while overexpression of SCD1 attenuated the effect of GluOC. Overall, the results of this study suggest that GluOC decreases SCD1 by activating AMPK to alleviate hepatocyte lipid accumulation. Furthermore, we also demonstrate that GPRC6A is the receptor for GluOC in HepG2 cells, which provides a new target for improving NAFLD in further research.

## Figures and Tables

**Table 1:** Interaction type of GluOC and GPRC6A.

CHAIN A	Residue	CHAIN B	Residue	Interaction type
GluOC	Tyr-38	GPRC6A	Ile-765	Hydrogen
GluOC	Tyr-42	GPRC6A	Lys-772	Hydrogen

**Table 2:** Primers for RT–qPCR.

Gene	Species	Forward Primer (5′→3′)	Reverse Primer (5′→3′)
FAS	Human	GGGATGAACCAGACTGCGTG	TCTGCACTTGGTATTCTGGGT
ACC1	Human	ATGTCTGGCTTGCACCTAGTA	CCCCAAAGCGTAACAAATTCT
SCD1	Human	AGCTCATCGTCTGTGGAGCC	GCCACGTCGGGAATTATGAGG
SREBP-1c	Human	CACCGTTTCGTGGATGG	CCCGCAGCATCAGAACAGC
GAPDH	Human	TGCACCACCAACTGCTTAGC	GGCATGGACGGTCATGAG
FAS	Mouse	GCTGCGGAAACTTCAGGAAAT	AGAGACGTGTCACTCCTGACTT
ACC1	Mouse	GAGGTACCGAAGTGGCATCC	GTGACCTGAGCGTGGGAGAA
SCD1	Mouse	TTCTTCTCTCACGTGGGTTG	CGGGCTTGTAGTACCTCCTC
SREBP-1c	Mouse	GTGAGCCTGACAAGCAATCA	GGTGCCTACGCGGCAAGAG
β-actin	Mouse	GATCTGGCACCACACCTTCT	GGGGTGTTGAAGGTCTCAAA

## Data Availability

The data presented in this study are available on request from the corresponding author.

## Sample Availability

Samples of the compounds are available from the authors.
